# Role of Chitinase 3–like‐1 in Myelofibrosis via Fibroblast‐Produced Extracellular Matrix Enhancement

**DOI:** 10.1002/jcp.70166

**Published:** 2026-04-06

**Authors:** Shoichiro Kato, Toshikuni Kawamura, Takaaki Maekawa, Hiraku Ogata, Noriaki Tachi, Yukiko Osawa, Shinichi Kobayashi

**Affiliations:** ^1^ Division of Hematology, Department of Internal Medicine National Defense Medical College Tokorozawa Saitama Japan; ^2^ Division of Palliative Care National Defense Medical College Hospital Tokorozawa Saitama Japan; ^3^ Division of Biochemistry, Department of Biomedical Sciences Nihon University School of Medicine Oyaguchi Kami‐cho Tokyo Japan

**Keywords:** chitinase 3‐like 1, fibrocyte, myelofibrosis, myeloproliferative leukemia, myeloproliferative neoplasm, secondary myelofibrosis, splenomegaly

## Abstract

Cluster of differentiation 14^+^ monocytes produced spindle‐shaped fibrocytes, similar to fibroblasts. Recent studies have reported that fibrocytes are crucial for the development and progression of primary myelofibrosis (MF); however, their functional role remains unclear. We compared monocytes and fibrocytes using RNA sequencing for gene expression profiles. We focused on chitinase 3–like‐1 (CHI3L1), which causes inflammation, organ fibrosis, and extracellular tissue remodeling. We found higher CHI3L1 levels in patients with myeloproliferative neoplasm with MF than in those without MF. Further, serum CHI3L1 levels were significantly associated with the presence of MF and splenomegaly in patients with lymphoid tumors. Romiplostim‐induced MF in a mouse model demonstrated extensive bone marrow (BM) *CHI3L1* mRNA expression, which was reversed by clodronate treatment. Two MF induction experiments on *CHI3L1* ^*−/−*^ mice, based on romiplostim or Janus kinase 2 mutations, revealed fewer reticular fibers in silver‐stained BM slices than in wild‐type mice. A culture assay revealed that high CHI3L1 concentrations promoted extracellular matrix production by fibroblast cell lines, and that a CHI3L1‐neutralizing antibody abrogated this effect. These results indicate the importance of CHI3L1 in the association between fibrocytes and fibroblasts in MF and could be a focus for future treatment.

## Graphical abstract

Our results indicate the involvement of CHI3L1 in the fibrocyte–fibroblast interaction and exacerbate BM fibrosis by promoting extracellular matrix formation. CHI3L1 is a missing link between fibrocytes and myofibroblasts and is a new potential therapeutic target for MF treatment.

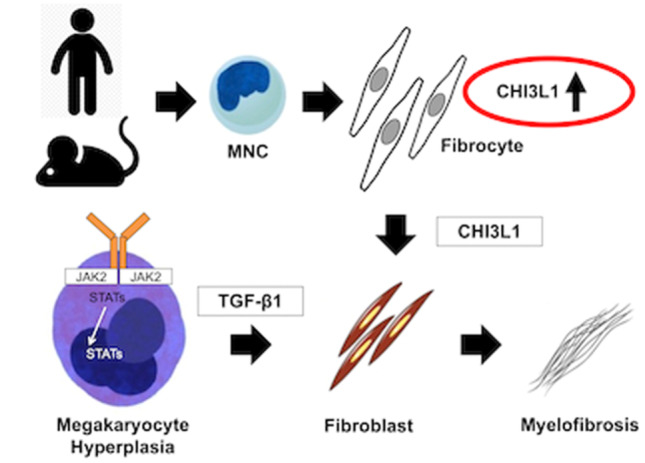

## 1 Introduction

Myelofibrosis (MF) is a crucial pathological feature of primary MF (PMF), post‐essential thrombocythemia (ET) MF, and post‐polycythemia vera (PV) MF. MF forms are associated with driver mutations, Janus kinase (JAK) 2 (*JAK2*), calreticulin (*CALR*), and myeloproliferative leukemia (*MPL*) (thrombopoietin [TPO] receptor [TPO‐R]) (Tefferi et al. 2014). These driver mutations activate the JAK/signal transducers and activators of transcription signaling pathway, resulting in the growth and differentiation of a myeloproliferative neoplasm (MPN) clone. Simultaneously, TPO‐R signaling activation induces collagen production in myofibroblasts due to cytokine overproduction, such as transforming growth factor (TGF)‐β1 from platelets and megakaryocytes of MPN clones (Wagner‐Ballon et al. 2007). We previously revealed that TPO‐R signaling activation directly induces fibrocyte differentiation of MPN clones, causing MF in MPN (Maekawa et al. 2017).

Cluster of differentiation (CD) 14^+^ monocytes differentiate into spindle‐shaped fibroblast‐like blood cells, also known as fibrocytes, which express stromal (collagen I, procollagen, and collagen III) and hematopoietic (CD45, CD34, CD11b, and CD68) cell markers (Maekawa et al. 2017; Verstovsek et al. 2016), and are well known for their significant role in the pathologic fibrosis of various organs besides the bone marrow (BM). Recently, some studies revealed the essential role of fibrocytes in MF using animal models: murine xenograft model (Verstovsek et al. 2016), romiplostim (Rom, a TPO‐R agonist)‐induced murine model (Maekawa et al. 2017), and *JAK2*V617F transgenic mouse model (Ozono et al. 2021). Fibrocytes generate some collagen, and murine *JAK2*V617F fibrocytes expressed more TGF‐ β1 than normal fibrocytes (Ozono et al. 2021). However, the difference in TGF‐ β1 production between normal and *JAK2*V617F human fibrocytes remains unknown. Further, the molecular contribution of fibrocytes to MF remains unclear.

Fibrocytes expressed more chitinase 3–like‐1 (CHI3L1) than monocytes, as the RNA sequencing‐based gene profiles indicated. The 18‐glycosyl hydrolase‐related molecule, CHI3L1, is a member of the enzymatically inactive chitinase‐like protein family and is known as BRP‐39 in mice and YKL‐40 in humans (Lee et al. 2011; Riabov et al. 2014). CHI3L1 is expressed in various cells, such as neutrophils, macrophages, chondrocytes, endothelial cells, and tumor cells (Lee et al. 2009; Bussink et al. 2007; Funkhouser and Aronson 2007), and plays crucial roles in organ immune response, inflammation, remodeling, and fibrosis (Lee et al. 2009; Recklies et al. 2002; Knudsen et al. 2006; Johansen 2006). Furthermore, higher serum CHI3L1 levels were more prominent in patients with PMF than in those with PV and ET; however, their pathological significance is unknown (Bjørn et al. 2014).

This study evaluated the association between CHI3L1 and human fibrocytes and measured serum CHI3L1 levels in patients with MPN. Further, the roles of CHI3L1 were confirmed using the Rom‐induced MF, *CHI3L1*‐deficient mice, and *JAK2*V617F TG mouse models.

## 2 Materials and Methods

### 2.1 Human Subjects

This study complied with the National Defense Medical College Ethics Committee–approved protocols (Approval Numbers 2153 and 4419). This study enrolled 52 patients diagnosed with MPN between January 2017 and December 2018 and 80 patients with lymphoid tumors (plasma cell tumors or malignant lymphoma), either newly diagnosed or with recurrence, between August 2021 and August 2023. All diagnoses were established according to the 2016 WHO classification criteria (Arber et al. 2016), and written informed consent was obtained from all participants. In MPN patients, one pathologist and one hematologist independently evaluated the severity of BM fibrosis based on the European consensus criteria (Thiele et al. 2005). MF‐0 represented patients with MPN without MF, and MF‐1, ‐2, or ‐3 represented those with MPN with MF. Patients with lymphoid tumors were evaluated for MF grade, presence of splenomegaly, and proportion of fibrocyte precursor cells, and serum CHI3L1 levels were assessed (supporting Table 1). We obtained blood samples from all patients and 17 healthy donors to measure serum CHI3L1 levels in a cross‐sectional manner, regardless of treatment stage. Peripheral blood samples from four healthy donors and three patients with *JAK2*V617F were used to culture human fibrocytes.

### 2.2 Human Fibrocyte Culture Assay

Density centrifugation from 10 mL of peripheral blood samples using Pancoll (PAN‐Biotech, Aidenbach, Germany) was used to purify peripheral blood mononuclear cells (PBMCs) from healthy donors. The PBMCs were cultured on 10‐cm culture dishes in 10 mL of Dulbecco's modified eagle medium (DMEM) (Wako 048‐33575, high glucose with sodium pyruvate and without additional amino acids; Wako, Osaka, Japan), with 2 mM of l‐glutamine (Nacalai tesque, Kyoto, Japan), 20% fetal bovine serum (FBS) (Corning, Corning, NY), 100 U/mL of penicillin, and 100 μg/mL of streptomycin (Thermo Fisher Scientific, Waltham, MA). The dishes were incubated at 37°C in a humidified incubator containing 5% CO_2_. After incubation for 3 days, the supernatant was removed to deplete the floating cells in the dishes, and DMEM was added again. The culture medium was changed every 4 days.

RNA sequencing analysis of fibrocytes to investigate the changes in gene expression profiles associated with fibrocyte differentiation used three sample types: adherent cells of days 2, 9, and 12, identified as monocytes, fibrocytes (day 9), and fibrocytes (day 12), respectively.

Trypsin‐EDTA solution (0.25%; Wako, Osaka, Japan) was added on days 4, 7, and 10 after PBMCs began to emerge in culture. Cells were dislodged, and 2 × 10^5^ cells/well were cultured for 48 h on a 6‐well plate (Becton, Dickinson and Company, Franklin Lakes, USA). They were recognized as fibrocytes (day 6), fibrocytes (day 9), and fibrocytes (day 12). A previous report (Pilling et al. 2009) revealed that an elongated spindle‐shaped adherent cell with an oval nucleus indicated differentiated fibrocytes. Each well was counted in five low‐power fields of view. Cell lysates and supernatants were collected to investigate the expression of *CHI3L1* mRNA, CHI3L1 protein, and TGF‐β protein and to perform a culture assay with the human fibroblast cell line HS‐5 (species: *Homo sapiens*; sex: male; tissue of origin: fibroblast; official cell line name: HS‐5, RRID: CVCL_3720; ATCC CRL‐3611, Manassas, VA, USA) (Roecklein and Torok‐Storb 1995).

HS‐5 cells were cultured in DMEM (ATCC 30‐2002, high glucose with sodium pyruvate, sodium bicarbonate, and amino acids) supplemented with 10% FBS, penicillin, and streptomycin. The culture medium was changed every 3 days.

### 2.3 RNA Sequencing Analysis of Human Monocytes and Fibrocytes

We used the RNeasy Mini Kit (QIAGEN, Hilden, Germany) to extract the total RNA from monocytes, fibrocytes (day 9), and fibrocytes (day 12), following the manufacturer's instructions. The Bioanalyzer 2100 RNA Nano 6000 Assay Kit (Agilent Technologies, CA, USA) was used to evaluate RNA integrity. We utilized 1% agarose gel to monitor RNA degradation and contamination. A Hiseq. 4000 (Illumina, San Diego, USA) generated 150‐bp paired‐end reads for total RNA sequencing. CLC Genomics Workbench 11.0 (QIAGEN) was used to statistically analyze RNAseq data, and the human hg19 reference genome and annotation data were retrieved from the Ensembl website (http://asia.ensembl.org/info/data/ftp/index.html). Each pairwise comparison between the three groups was analyzed for differential gene expression. A false discovery rate of < 0.05 and a log2‐fold change cut‐off of 2 were applied. The differentially expressed gene list was further filtered to include genes with absolute fragments per kilobase of exon per million mapped reads (FPKM) values of > 1 in each group to generate heatmaps. The map was drawn after extracting 25 genes that exhibited high variability.

### 2.4 Luminex Assay

Serum samples of patients with MPN were stored at −20°C until analysis. Human Luminex Assays (R&D Systems, Minneapolis, MN) were used to evaluate CHI3L1 serum levels, as previously described (Maekawa et al. 2019).

### 2.5 Mouse Model of MF Induced by a TPO‐R Agonist

Eight‐week‐old female C57BL/6 J mice (CLEA Japan, Tokyo, Japan) were classified into two groups: control (*n* = 16) and Rom (*n* = 16). Each group was further categorized into three: treated once (*n* = 4), treated twice (*n* = 8), and treated thrice (*n* = 4) groups. The control group mice were administered saline, and the Rom group mice were subcutaneously injected with 1 mg/kg of Rom at the neck on days 1, 8, and 15. Peripheral blood was drawn by tail cutting or cardiac puncture. Femurs, spleens, and tibias were extracted on day 15 after euthanasia for the twice‐treated groups. As previously described (Maekawa et al. 2017), spleens were weighed, and the cells were isolated using a 40‐μL cell strainer (BD Biosciences, San Jose, USA). RPMI‐1640 (Sigma‐Aldrich, St. Louis, USA) was used to flush tibia‐derived BM cells. Spleen and BM cells were hemolyzed following the manufacturer's (BD Pharm Lyse, BD Bioscience) instructions. A standard protocol was used for the morphologic analysis of mouse femur‐derived BM samples stained with hematoxylin/eosin and silver, as previously described (Maekawa et al. 2017). Furthermore, mouse BM samples were immunostained with an anti‐α‐smooth muscle antigen (SMA) antibody (DAKO, Tokyo, Japan), as previously described (Maekawa et al. 2017).

Eight‐week‐old female C57BL/6 J mice were then classified into three groups: control (*n* = 6), Rom (*n* = 8), and Rom + clodronate liposomes (CLs) (Formumax, Sunnyvale, CA, USA) (*n* = 6). The mice in the Rom and Rom + CLs groups were administered 1 mg/kg of Rom, and those in the control group were administered saline on days 1 and 8. The Rom + CLs group mice received 200 μL of CLs intraperitoneally on day 1 and 100 μL on days 3, 7, 11, and 14. Similarly, the control and Rom groups were administered control liposomes (Formumax). Tibial‐ and femoral‐derived BMs were extracted from all three groups after euthanasia on day 15 and treated as previously described (Maekawa et al. 2017).

### 2.6 *CHI3L1* Knockout Mice Preparation

*CHI3L1* ^*−/−*^ mice (C57BL/6 background) were previously prepared in our laboratory (Higashiyama et al. 2019). Rom of 1 mg/kg was administered to 8‐week‐old wild‐type female C57BL/6 (*n* = 19) and *CHI3L1* ^*−/−*^ (*n* = 21) mice on days 1 and 8. Spleens, tibias, and femurs were collected after euthanasia on day 15 and treated as described above. Additionally, 1 mg/kg of Rom was administered to 8‐week‐old wild‐type female C57BL/6 (*n* = 4) and *CHI3L1* ^*−/−*^ (*n* = 6) mice on days 1, 8, and 15. The spleens, tibias, and femurs were collected and treated similarly after euthanasia on day 22.

### 2.7 Preparation of *JAK2*V617F Transgenic (TG) Mice

Dr. Shimoda (Miyazaki University, Miyazaki, Japan) kindly provided *JAK2*V617F TG mice (Ozono et al. 2021). We created *JAK2*V617F/*CHI3L1* ^−/−^ mice by crossing *JAK2*V617F TG mice with *CHI3L1* ^−/−^ mice. Spleens, tibias, and femurs of 12‐week‐old (*n* = 5) *JAK2*V617F TG mice and *JAK2*V617F/*CHI3L1* ^−/−^ mice (*n* = 7) were collected after euthanasia on day 15 and treated as described above. We used HALO AI (Indica Labs, Corrales, NM, USA) (Kawamura et al. Forthcoming) to develop deep learning–based fibrosis quantification to assess fibrosis in these mouse groups.

### 2.8 Addition of Supernatant Obtained from Cultured Fibrocytes and Neutralizing Antibody to HS‐5

HS‐5 cells were incubated at 2 × 10^5^ cells/well for 24 h in 6‐well plates. The fibrocyte supernatant (days 6, 9, and 12) was added to each plate as a culture medium. We evaluated the effect of anti‐CHI3L1 antibody (10 μg/mL, Merck Millipore, Burlington, MA, USA) (Faibish et al. 2011) on HS‐5 cell culture with day 12 fibrocyte supernatant. Mouse monoclonal IgG1κ was used as a negative control. The cell lysate was collected to analyze collagen mRNA expression after 48 h.

### 2.9 Enzyme‐Linked Immunosorbent Assay Analysis of Supernatant From Human Fibrocytes and Mouse Serum Samples

The human fibrocyte supernatant was evaluated using human CHI3L1 and TGF‐β Quantikine ELISA Kit (R&D systems), and mouse serum samples were measured using mouse CHI3L1 Quantikine ELISA Kit (R&D systems), following the manufacturer's instructions.

### 2.10 Analysis of mRNA Expression in Mouse and Human Samples

Total cellular RNA from human fibrocytes, mouse spleen, mouse BM, and HS‐5 cells were obtained using RNeasy Mini Kit (QIAGEN) following the manufacturer's instructions. The SuperScript^TM^ III First‐Strand Synthesis SuperMix for qRT‐polymerase chain reaction (PCR) (Invitrogen, Carlsbad, CA) was used. The Light Cycler 480 SYBR Green I Master (Roche, Basel, Switzerland) was used to perform quantitative real‐time PCR, as described by the manufacturer. Supporting Table 2 lists the primer sequences used in this study. The mRNA values were analyzed using comparative CT (2^−ΔΔCT^) and normalized with the mRNA levels of encoding glyceraldehyde‐3‐phosphate dehydrogenase (GAPDH).

### 2.11 Statistical Analysis

Table 1 shows the variables obtained from human data. The JMP Pro 12 software (SAS Institute, Cary, NC) was used for all analyses. We used Fisher's exact and Mann–Whitney *U* tests to compare the characteristics of patients with MPN MF(−) and MPN MF(+).

**Table 1:** Characteristics of Patients with Myeloproliferative Neoplasm (MPN) According to Myelofibrosis (MF).

Variables	Number of patients (%)		*p* value
	MPN MF(−)	MPN MF(+)	
	(*n* = 21)	(*n* = 29)	
Age (years); median (range)	65 (24–85)	69 (40–86)	0.491
Gender			1
Male	9 (42.9%)	12 (41.4%)	
Female	12 (57.1%)	17 (58.6%)	
Hemoglobin, g/dL; median (range)	14.2 (11.1–18.6)	12.4 (6.0–18.8)	0.059
Leukocytes, ×10^9^/L; median (range)	8.7 (5.3–17.5)	14.7 (2.8–60.3)	0.09
Platelets, ×10^9^/L; median (range)	56.3 (17.0–130.7)	62.3 (12.5–176.7)	0.852
Genetic mutation			0.522
* JAK2*	12 (57.1%)	16 (55.2%)	
* CALR*	2 (9.5%)	6 (20.7%)	
* MPL*	0 (0.0%)	1 (3.4%)	
Triple‐negative	7 (33.3%)	6 (20.7%)	
Primary disease			0.113
PV	8 (38.1%)	11 (37.9%)	
ET	12 (57.1%)	11 (37.9%)	
PMF	1 (4.8%)	7 (24.2%)	
MF grade			
MF‐0	21 (100.0%)	0	
MF‐1	0	15 (51.7%)	
MF‐2	0	8 (27.6%)	
MF‐3	0	6 (20.7%)	
Medication			
Hydroxyurea	10 (47.6%)	8 (27.6%)	0.232
Anagrelide	0 (0.0%)	1 (3.4%)	1
No medication	11(52.4%)	20 (69.0%)	0.891
Splenomegaly	0 (0.0%)	11 (37.9%)	**0.001** a
LDH, U/L; median (range)	214.5 (149–291)	395.7 (151–1945)	**0.013**
CRP, mg/dL; median (range)	0.36(0.3–0.9)	1.43 (0.3–20.1)	0.077
CHI3L1, ng/mL; median (range)	29.0 (4.7–108.3)	57.3(6.0–187.1)	**0.003**

Abbreviations: *CALR*, calreticulin; CHI3L1, chitinase 3–like‐1; CRP, C‐reactive protein; EP, post‐essential thrombocythemia; *JAK2*, Janus activating kinase 2; LDH, lactate dehydrogenase; MF, myelofibrosis; *MPL*, myeloproliferative leukemia; MPN, myeloproliferative neoplasm; PV, post‐polycythemia vera.

^a^ Bold formatting indicates significant *p* values.

The remaining human and mouse data are expressed as means ± standard error of the mean. One‐way analysis of variance with *post hoc* Tukey's test was used to analyze data from more than two groups, while the Mann–Whitney *U* test was utilized to compare data between two groups. The JMP Pro 12 software (SAS Institute) and GraphPad Prism 10 (GraphPad Software, La Jolla, CA) were used for data analysis and plotting. All probability (P) values were two‐sided, and statistical significance was taken at *p* < 0.05.

## 3 Results

### 3.1 Human Fibrocyte Differentiation Correlates With an Increase in CHI3L1 Expression

**Figure 1:**
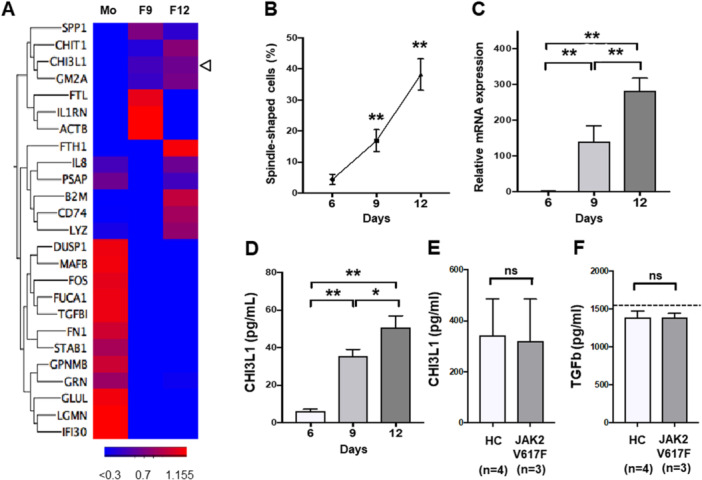
Significant increase in CHI3L1 expression associated with fibrocyte differentiation. (A) Heatmap of RNA sequencing analysis of human monocytes and fibrocytes. The list of differentially expressed genes was further filtered for genes with an absolute FPKM value of > 1 in each group, and a heatmap was generated. After extracting 25 genes that exhibited high variability, a map was drawn. (B) Human fibrocyte culture assay. The ratio of spindle‐shaped cells increased with culture time. (C) Quantitative real‐time polymerase chain reaction (PCR) of human fibrocytes. *CHI3L1* mRNA expression increased with fibrocyte differentiation. (D) Enzyme‐linked immunosorbent assay (ELISA) analysis of human fibrocytes. CHI3L1 protein levels correlated with the degree of mRNA expression. (E, F) ELISA analysis of human fibrocyte culture supernatants. The amount of CHI3L1 and TGF‐β1 produced by fibrocytes collected from patients positive for *JAK2*V617F was comparable to that from healthy donors. *p < 0.05, **p < 0.01.

RNA sequencing analysis identified 13 genes whose expression was significantly higher in fibrocytes than in monocytes. The *CHI3L1* gene demonstrated an increased expression associated with fibrocyte differentiation (Figure 1A). In contrast, fibrocyte differentiation decreased TGF‐β1 expression. The frequency of spindle‐shaped fibrocytes increased as culture days progressed (on days 6, 9, and 12, *p* < 0.01) (Figure 1B), and *CHI3L1* mRNA expression and CHI3L1 protein levels in the supernatant increased with fibrocyte differentiation (*p* < 0.01) (Figure 1C,D). CHI3L1 and TGF‐β1 levels produced by fibrocytes collected from three PV patients positive for *JAK2*V617F (the *JAK2* allele burden in the PB samples was 90.8%, 31.8%, and 94.2%, respectively) were comparable to those from four healthy donors (Figure 1E,F).

### 3.2 Elevated CHI3L1 Serum Levels Are Associated With the Degree of MF

Table 1 shows patient characteristics and the results of data analysis. Patients with MPN were categorized into two groups: those without MF (*n* = 21) and those with MF (*n* = 29). The primary diseases were PV (*n* = 19), ET (*n* = 23), and PMF (*n* = 8). The types of genetic mutations were *JAK2* (*n* = 28), *CALR* (*n* = 8), *MPL* (*n* = 1), and triple‐negative (*n* = 13). Hydroxyurea was administered to 18 patients and anagrelide to 1 patient during sample collection. Eleven patients demonstrated palpable splenomegaly.

**Figure 2:**
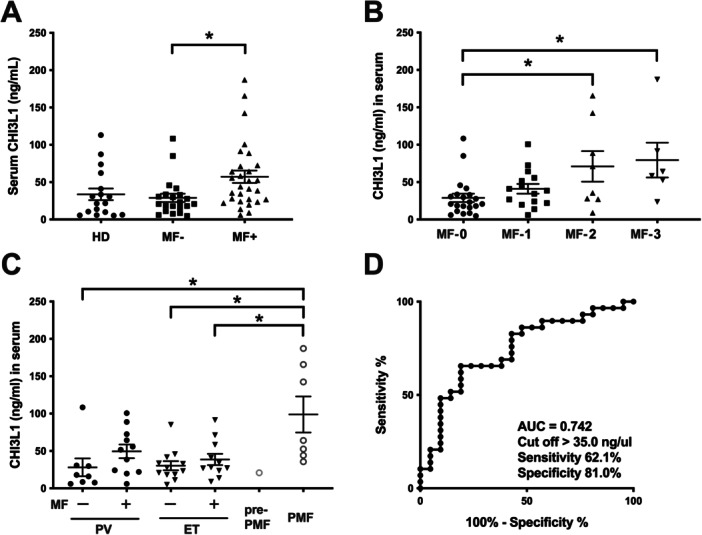
Serum CHI3L1 levels were elevated in patients with MPN with MF. (A) Comparison of serum CHI3L1 levels between healthy donors and patients with MPN. Serum CHI3L1 levels in patients with MPN with MF (*n* = 29) were significantly higher than those in patients with MPN without MF (*n* = 21). (B) Subgroup analysis of serum CHI3L1 levels by MF grade. Serum CHI3L1 levels increased in association with MF grade, and levels in patients with MF‐2 and MF‐3 were higher than those in patients with MF‐0. (C) Subgroup analysis of serum CHI3L1 levels by MPN type. Significantly higher serum CHI3L1 levels were observed in patients with PMF than in those with PV without MF or ET. (D) ROC curve showing the cut‐off value for serum CHI3L1 concentration for separating patients with MPN with MF from those without MF. The AUC was 0.742, and the sensitivity and specificity identifying MF were 62.1% and 81.0%, respectively, when the cut‐off was set at a serum CHI3L1 concentration of > 35.0 ng/mL. *p < 0.05.

The median serum CHI3L1 levels in healthy donors, patients with MPN without MF, and patients with MPN with MF were 28.2, 29.0, and 57.3 ng/mL, respectively (Figure 2A). Patients with MPN with MF demonstrated a higher incidence of splenomegaly (*p* < 0.001) and higher serum LDH (*p* = 0.013) and CHI3L1 (*p* = 0.003) levels than patients with MPN without MF. CHI3L1 serum levels increased in association with MF grade (Figure 2B). Subgroup analysis by MPN type revealed significantly higher serum CHI3L1 levels in patients with PMF than in those with PV without MF and ET. In contrast, no apparent differences were observed between other MPNs with and without MF (Figure 2C). We created receiver operating characteristic (ROC) curves to determine the cut‐off serum CHI3L1 value for distinguishing patients with MPN with MF from those without MF. The resulting area under the curve was 0.742, and the sensitivity for identifying MF and specificity were 62.1% and 81.0%, respectively, when the cut‐off was set at a serum CHI3L1 concentration of > 35.0 ng/mL (Figure 2D). In patients with lymphoid tumors, serum CHI3L1 levels significantly were higher in the presence of myelofibrosis (MF‐1 or higher), splenomegaly, and an increased proportion of fibrocyte precursor cells (defined > 8.4% as high) (Supporting Figure 1).

### 3.3 Elevated *CHI3L1* mRNA Expression in BM of the Rom‐Induced Murine MF Model Abrogated via Clodronate Treatment

**Figure 3:**
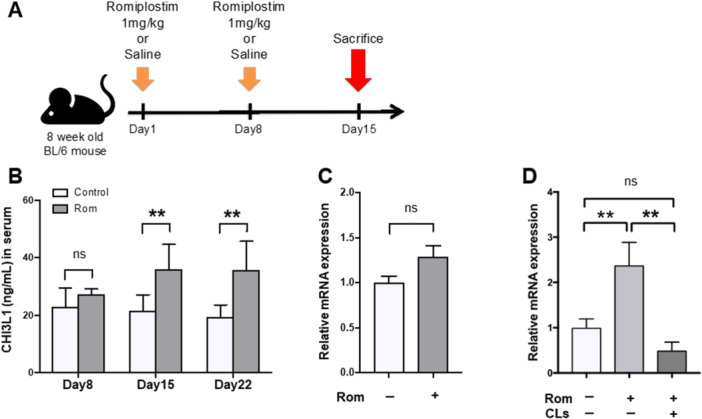
Increased *CHI3L1* mRNA expression was associated with MF progression, which was abrogated by CLs. (A) The protocol for the mouse model of MF induced by a TPO‐R agonist. (B) Serum CHI3L1 concentrations of both control and Rom groups. Serum CHI3L1 levels in control mice (*n* = 4) treated once did not differ from the Rom (*n* = 4) group. However, serum CHI3L1 levels in mice treated twice (*n* = 8) and thrice with Rom (*n* = 4) were significantly higher than those in mice treated with saline twice (*n* = 8) and thrice (*n* = 4). (C) Relative mRNA expression analysis in the spleen obtained from mice treated with saline or Rom twice. The Rom and control groups demonstrated no difference in the expression levels in the spleen. (D) Analysis of relative mRNA expression in BM in the control, Rom, and Rom + CLs groups. *CHI3L1* mRNA expression levels in BM were higher in the Rom group (*n* = 8) than in the control group (*n* = 6). CL treatment abrogated this effect (*n* = 8). *p < 0.05, **p < 0.01.

We evaluated CHI3L1 serum and mRNA expression levels in the spleen and BM using the Rom‐induced murine model of MF to confirm the dynamics of MF (Figure 3A). Serum CHI3L1 levels in mice treated with Rom were more than twice as high as those in the control group (*p* < 0.05) (Figure 3B). *CHI3L1* mRNA levels in the spleen showed no apparent difference between the control and Rom groups (Figure 3C). In contrast, the BM of the Rom group demonstrated higher *CHI3L1* mRNA expression levels than that of the control group (*p* < 0.01) (Figure 3D). As previously reported (Maekawa et al. 2017), CL administration eliminated monocytes, macrophages, and fibrocytes to alleviate BM fibrosis in this model. We confirmed the effect of CLs on MF and revealed that clodronate treatment abrogated the elevation of *CHI3L1* mRNA expression in the BM of the Rom group (*p* < 0.01) (Figure 3D).

### 3.4 *CHI3L1* ^*−/−*^ Mice Demonstrated Decreased *Col3a1* mRNA Expression and Fewer Reticular Fibers in the BM

**Figure 4:**
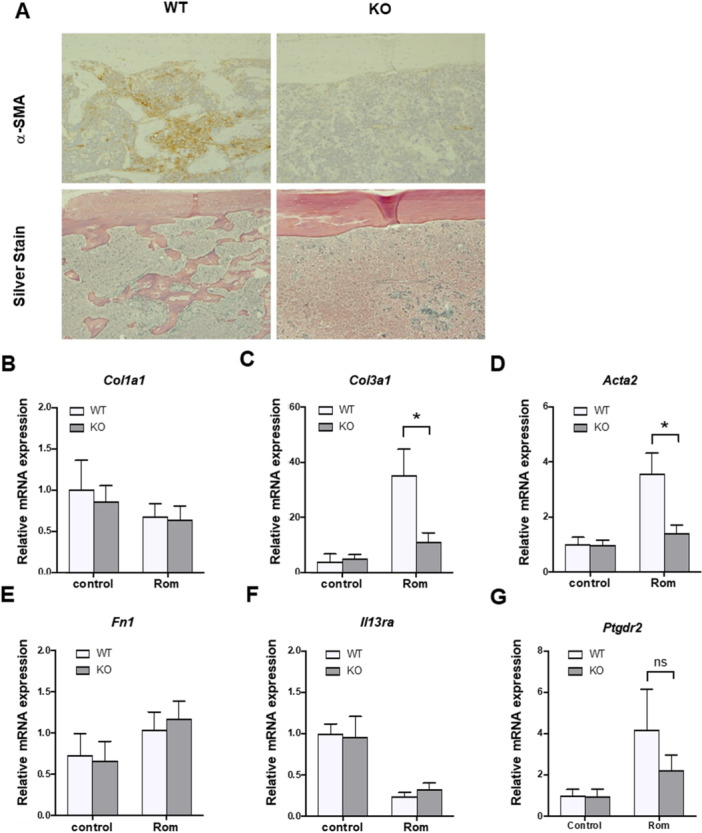
*CHI3L1* ^*−/−*^ mice demonstrated fewer reticular fibers and decreased collagen factor mRNA levels. (A) Silver staining and immunohistochemical staining with α‐smooth muscle actin (α‐SMA) of a femur‐derived BM section from wild‐type and *CHI3L1* ^−/−^ mice treated with Rom. The BM section of wild‐type mice is shown to the left. Silver staining of the BM stroma revealed dense reticular fibers with extensive intersections, and the BM stroma was diffusely α‐SMA positive and stained brown by the α‐SMA staining. The image represents 19 wild‐type mice with an MF‐2 grade. The BM section of *CHI3L1* ^*−/−*^ mice is shown to the right. Fewer reticular fibers and α‐SMA‐positive areas were observed and compared with wild‐type mice. The image represents 21 *CHI3L1* ^−/−^ mice, and the MF grade was evaluated as MF‐1. (B–G) Analysis of relative mRNA expression in BM obtained from wild‐type and *CHI3L1* ^*−/−*^ mice treated with saline or Rom twice. *CHI3L1* ^−/−^ mice demonstrated significantly decreased *COL3A1* and *Acta2* mRNA expression. Meanwhile, *COL1A1*, *Fn1*, *IL‐13rα2*, or *Ptgdr2* demonstrated no obvious changes. Results are expressed as means ± standard error of the mean (SEM). The Mann–Whitney *U* test was used to evaluate statistical significance. *p < 0.05.

We studied the functional role of CHI3L1 in MF development using Rom‐treated wild‐type and *CHI3L1* ^−/−^ mice. Silver and immunohistochemical staining with α‐SMA of the femur BM sections revealed that *CHI3L1* ^*−/−*^ mice presented significantly lower MF grade and positive staining area compared with the wild‐type mice on day 15 (Figure 4A, Table 2). However, hemoglobin, platelet counts, or spleen weight demonstrated no significant differences (Supplementary Figure 2). Gene expression analysis of fibrosis‐related genes in the BM demonstrated significantly decreased *Col3a1* and *Acta2* mRNA in *CHI3L1* ^*−/−*^ mice (*p* < 0.05) compared to the wild‐type mice, with no apparent difference in *Col1a1* and *Fn1* mRNA expression (Figure 4B–E). *CHI3L1* ^*−/−*^ and wild‐type mice demonstrated similar *Il‐13rα2* and *Ptgdr2* mRNA expression, which are CHI3L1 receptors (Figure 4F,G).

**Table 2:** Fewer Myelofibrosis Grade of *CHI3L1* ^−/−^ Mice Compared with Wild‐type Mice.

*MF grade*	Number of mice			
	Day15**		Day22*	
	WT	KO	WT	KO
	(*n* = 15)	(*n* = 15)	(*n* = 4)	(*n* = 6)
MF‐0	0	2	0	1
MF‐1	0	8	0	4
MF‐2	14	5	4	1
MF‐3	1	0	0	0

*Note:* The Mann–Whitney *U* test was used to evaluate the statistical significance of MF grade between WT and KO mice.

Abbreviations: KO, knockout; MF, myelofibrosis; WT, wild type.

* *p* < 0.05

** *p* < 0.01.

### 3.5 *JAK2*V617F/*CHI3L1* ^−/−^ Mice Had a Smaller Area of Reticular Fibers Than *JAK2*V617F TG Mice

**Figure 5:**
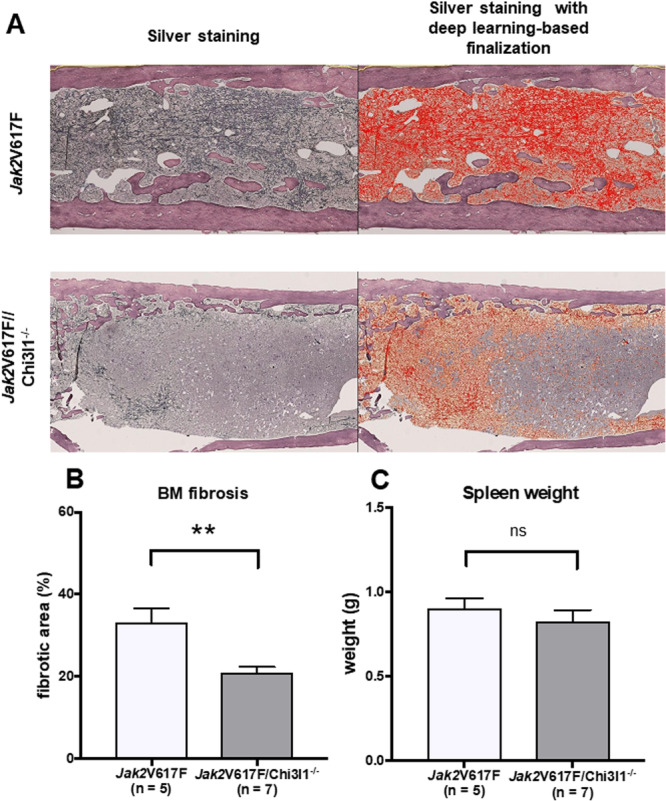
The areas of reticular fibers (RFs) in the BM of mice were measured using HALO AI. (A) Silver staining slides comparing *JAK2*V617F TG mice and *JAK2*V617F/*CHI3L1* ^−/−^ mice. Deep learning‐based finalization allowed the exclusion of normal BM to determine reticular fibers (red area) alone. Area Quantification v2 (HALO application) was used to measure RF area. (B) *JAK2*V617F/*CHI3L1* ^−/−^ mice demonstrated significantly reduced BM RF area compared with *JAK2*V617F TG mice. (C) Spleen weight between *JAK2*V617F TG mice and *JAK2*V617F/*CHI3L1* ^−/−^ mice exhibited no significant differences. Results are expressed as means ± SEM. The Mann–Whitney *U* test was used to evaluate statistical significance. *p < 0.05, **p < 0.01.

We induced MF using *JAK2*V617F transgenic mice to better understand the function of CHI3L1. Silver staining revealed a diffuse and dense increase in reticular fibers with extensive intersections in both *JAK2*V617F TG and *JAK2*V617F/*CHI3L1* ^−/−^ mice. However, silver staining revealed fewer collagen bundles, indicating an MF‐2 grade in both groups. The European consensus criteria‐based evaluation method demonstrated no obvious differences (data not shown). We used a novel deep learning–based method to quantify fibrosis (manuscript submitted), providing a more quantitative evaluation of reticular fibers between the two groups (Figure 5A). The mean reticular fiber area was significantly smaller in the *JAK2*V617F/*CHI3L1* ^*−/−*^ mice than in the *JAK2*V617F TG mice (*p* < 0.001) (Figure 5B), while spleen weight between *JAK2*V617F TG mice and *JAK2*V617F/*CHI3L1* ^−/−^ mice showed no significant differences (Figure 5C).

### 3.6 CHI3L1 Is Involved in the Interaction Between Fibrocytes and Human Fibroblast Cell Line HS‐5, as Evidenced by Coculture Assays

**Figure 6:**
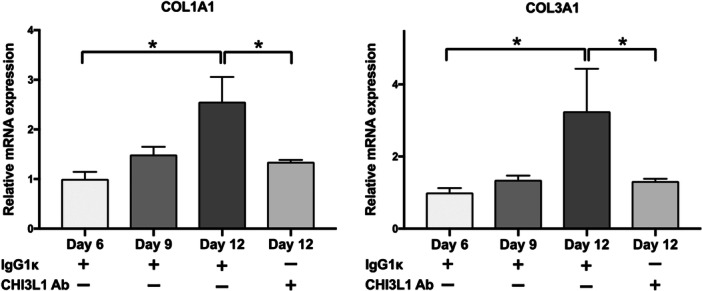
CHI3L1 produced by fibrocytes stimulated human fibroblasts to produce extracellular matrix components. (A, B) *COL1A1* and *COL3A1* mRNA expression were significantly higher in HS‐5 cells cultured with day 12 fibrocyte supernatants than in those cultured with day 6 and day 9 fibrocyte supernatants. Moreover, *COL1A1* and *COL3A1* mRNA expression were significantly lower in cultures treated with the CHI3L1‐neutralizing antibody than in those treated with a mouse monoclonal IgG1κ control antibody. Data are expressed as means ± standard error of the mean. *p < 0.05, analysis of variance with Tukey's post hoc test.

We used the human fibroblast cell line HS‐5 to assay human fibrocytes and fibroblasts by noncontact coculture to assess how CHI3L1 interacts with fibroblasts. Based on the finding that fibrocytes secrete increased CHI3L1 concentrations with culture time, we prepared three types of supernatants (days 6, 9, and 12) as a culture medium for HS‐5. Culturing HS‐5 cells with fibrocyte supernatant significantly increased the mRNA expression of *COL1A1* and *COL3A1* (*p* < 0.05), indicating an association with fibrocyte differentiation. The CHI3L1‐neutralizing antibody abrogated these increases, indicating that CHI3L1 secreted from fibrocytes is crucial for fibroblast extracellular matrix production (Figure 6).

## 4 Discussion

Some studies have indicated the involvement of CHI3L1 in lung, liver, and kidney fibrosis. High serum CHI3L1 levels were associated with disease progression in patients with idiopathic pulmonary fibrosis, reflecting an attempt to diminish injury and repair and contribute to lung fibrosis (Zhou et al. 2014). Montgomery et al. (2017) revealed that CHI3L1 activates myofibroblasts, thereby promoting renal fibrosis in a mouse model of ischemia‐reperfusion injury. Urine CHI3L1 level was associated with a more significant estimated glomerular filtration rate decrease and progressive renal fibrosis (Puthumana et al. 2021). CHI3L1 is a known biomarker of the degree of liver fibrosis and extracellular matrix synthesis (Johansen et al. 2000) and is a profibrogenic factor that is overexpressed in the aging liver and patients with liver fibrosis (Nishimura et al. 2021). Liver macrophages produced CHI3L1, which inhibited their apoptosis in a mouse liver fibrosis model (Higashiyama et al. 2019). Meanwhile, CHI3L1 has recently been implicated in hematologic disorders, with elevated CHI3L1 expression associated with lymphoma onset, progression, severity, and poor prognosis (Wang et al. 2025). Furthermore, CHI3L1 concentration in the serum of multiple myeloma patients was closely correlated with disease severity and high concentration CHI3L1 predicted the progression of multiple myeloma disease (Tang et al. 2025).

Our results corroborated *the role of* CHI3L1 as a profibrotic factor in BM fibrosis. CHI3L1 promotes BM fibrosis as a malignant process and induces tissue fibrosis as part of the repair after injury in other organ fibrosis. High serum concentrations of CHI3L1 have already been reported in patients with PMF, although the cause and role in pathophysiology remain unknown (Bjørn et al. 2014). Our analysis revealed an association between serum CHI3L1 concentration and BM fibrosis. The study of Bjørn et al. (2014) may have not contained sufficient patients with PV and ET with MF. Based on these results, our mouse experiments revealed an increased *CHI3L1* mRNA expression in the BM with the frequency of romiplostim administration and the degree of fibrosis in the Rom‐induced murine MF model. Further, CLs with fibrocyte removal suppressed CHI3L1 in BM and MF (Maekawa et al. 2017). Furthermore, we revealed that the presence of CHI3L1 contributes to fibrosis exacerbation in Rom‐induced murine MF models and *JAK2*V617F TG mice. The association between CHI3L1 and BM fibrosis was demonstrated in mice and humans with great significance.

Conversely, there is scope for further research on the CHI3L1‐secreting cells and the mechanisms of physiological interaction. CHI3L1 is produced by various cells, including macrophages, neutrophils, chondrocytes, vascular smooth muscle cells, and hepatic stellate cells. Macrophages demonstrated a strong expression of CHI3L1, recognized as a valuable marker of human macrophage differentiation in tissue, from immunohistochemical‐staining observations in organ fibrosis (Rehli et al. 1997; Rehli et al. 2003). Therefore, organ fibrosis associated with CHI3L1 is believed to mainly rely on macrophages (Higashiyama et al. 2019; Montgomery et al. 2017; Puthumana et al. 2021), which are named based on their tissue of origin. In the present study, CHI3L1 expression was higher in fibrocytes than in monocytes and macrophages. Moreover, CHI3L1 expression levels increased with fibrocyte differentiation. Further, CLs, which is an eliminator of macrophages and fibrocytes in the mouse model, abrogated the elevated CHI3L1 observed in Rom‐induced murine MF models (Maekawa et al. 2017). Taken together, these findings indicate the involvement of fibrocytes in CHI3L1 secretion and MF progression.

Constitutive JAK/signal transducers and activators of transcription signaling cascade activation is thought to produce BM fibrosis in PMF due to *JAK2*V617F and TGF‐β overproduction, which subsequently occurs (Jacobson et al. 1978; Rampal et al. 2014). Analyses of the *JAK2*V617F mouse model revealed that neoplastic fibrocytes played a crucial role in PMF BM fibrosis. Their differentiation depends on TGF‐β1 in an autocrine manner (Ozono et al. 2021). However, we could not confirm the difference in TGF‐β1 production between normal and *JAK2*V617F fibrocytes in human samples. Further, *JAK2* mutation did not affect CHI3L1 production from fibrocytes. Our results revealed the role of TGF‐β1 produced by fibrocytes in BM fibrosis and indicated that they both play crucial roles as myofibroblast stimulators. This is particularly evident with an increasing number of fibrocytes. CHI3L1 was reported to activate the TGF‐β1 pathway in hepatocellular carcinoma, although its signaling pathway was unclear (Qiu et al. 2018). These reports indicate that CHI3L1 and TGF‐β1 stimulate myofibroblasts at least additively. However, the absence of TGF‐β1 hyperproduction in human *JAK2*V617F fibrocytes raised the possibility of discrepancies between humans and mice in BM fibrosis pathophysiology.

## 5 Conclusions

Our results indicate that CHI3L1 is critically involved in fibrocyte–fibroblast interactions and exacerbates BM fibrosis by promoting extracellular matrix formation. CHI3L1 represents the missing link between fibrocytes and myofibroblasts and may serve as a novel therapeutic target for MF.

## Author Contributions

**Shoichiro Kato, Toshikuni Kawamura:** Conceptualization; Writing—original draft; Investigation; Data curation; Methodology; Visualization; Formal analysis; Project administration. **Takaaki Maekawa:** Conceptualization; Writing—review and editing; Funding acquisition; Data curation; Project administration; Investigation; Validation; Methodology; Formal analysis; Visualization. **Hiraku Ogata, Noriaki Tachi:** Investigation; Data curation; Resources. **Yukiko Osawa:** Investigation; Data curation; Resources; Project administration. **Shinichi Kobayashi:** Supervision.

## Ethics Statement

This study complied with the National Defense Medical College Ethics Committee–approved protocols (Approval Numbers 2153 and 4419).

## Consent

This study enrolled 52 patients diagnosed with myeloproliferative neoplasm from January 2017 to December 2018 after obtaining written informed consent.

## Conflicts of Interest

The authors declare no conflicts of interest.

## Supporting information

Supporting_data_R1.

## Data Availability

The datasets generated during and/or analyzed in the current study are available from the corresponding author on reasonable request.
