# Supplementary material for: Role of Chitinase 3–like‐1 in Myelofibrosis via Fibroblast‐Produced Extracellular Matrix Enhancement
Source: J Cell Physiol. 2026 Apr 6;241(4):e70166. doi: 10.1002/jcp.70166 (PMC13051415; doi:10.1002/jcp.70166)
Supplement: Supplementary file 1 — Supporting_data_R1. [file JCP-241-0-s001.docx]

**Supplementary Table 1**

| **Variables** | **Number of patients**  **(n = 80)** |
| --- | --- |
| **Female, n** | **43** |
| **Age (years), mean ± SD** | **71.2 ± 10.0** |
| **Disease, n**  **Plasma cell neoplasms**  **Mature B-cell neoplasms**  **Diffuse large B-cell lymphoma**  **Follicular lymphoma**  **Other B-cell lymphomas**  **Mature T cell and NK cell neoplasms**  **Hodgkin Lymphoma** | **15**  **37**  **8**  **12**  **5**  **3** |
| **Myelofibrosis grading, n (%)**  **MF-0**  **MF-1**  **MF-2** | **34**  **41**  **5** |
| **Splenomegaly, n (%)** | **14** |
| **Increase in the proportion of fibrocyte precursor cells (define >8.4% as high)** | **20** |
| ***CHI3L1* (ng/mL), mean ± SD** | **316.7 ± 385.2** |

**Supplementary Table 2**

Primer sequences used for mRNA analysis

| Human |  | sequence (5'-3') | |
| --- | --- | --- | --- |
| *CHI3L1* | forward | GATGTGACGCTCTACGGCAT | |
|  | reverse | TGATGAAAGTCCGGCGACTC | |
| *COL1A1* | forward | GAGGGCCAAGACGAAGACATC | |
|  | reverse | CAGATCACGTCATCGCACAAC | |
| *COL3A1* | forward | TTGAAGGAGGATGTTCCCATCT | |
|  | reverse | ACAGACACATATTTGGCATGGTT | |
| *GAPDH* | forward | TCTCTGCTCCTCCTGTTCGAC | |
|  | reverse | AGTTAAAAGCAGCCCTGGTGA | |
|  |  |  |  |
| Mouse |  | sequence (5'-3') | |
| *Chi3l1* | forward | CCAACACTGAGAGACGCACT | |
|  | reverse | GGCTGGACCTCCTTTGTGAA | |
| *Col1a1* | forward | GACGCATGGCCAAGAAGACA | |
|  | reverse | CCTCGGGTTTCCACGTCTC | |
| *Col3a1* | forward | CTGTAACATGGAAACTGGGGAAA | |
|  | reverse | CCATAGCTGAACTGAAAACCACC | |
| *Acta2* | forward | CCCAGACATCAGGGAGTAATGG | |
|  | reverse | TCTATCGATACTTCAGCGTCA | |
| *Fn1* | forward | ATGTGGACCCTCCTGATAGT | |
|  | reverse | GCCCAGTGATTTCAGCAAAGG | |
| *Il13rα2* | forward | ACCGAAATGTTGATAGCGACAG | |
|  | reverse | ACAATGCTCTGACAAATGCGTA | |
| *Ptgdr2* | forward | CTCAGTCCTGCTGCTTTCAGA | |
|  | reverse | GACTGATCGGGTGTGCTGAT | |
| *Gapdh* | forward | TGGAGAAACCTGCCAAGTATGAT | |
|  | reverse | TTGCTGTTGAAGTCGCAGGA | |

**Supplementary Figure 1**





**Supplementary Figure 2**


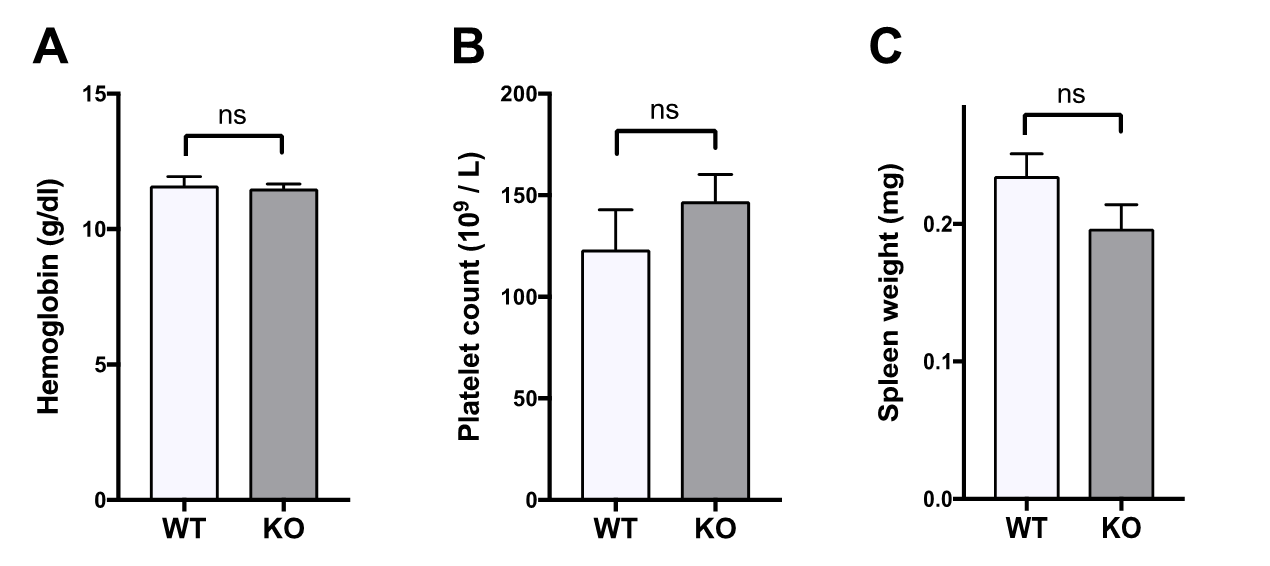


**Supplementary Figure Legends**

**Supplementary Figure 1.** In patients with lymphoid tumors, serum *CHI3L1* levels were significantly higher in the presence of myelofibrosis (MF-1 or higher) (A) splenomegaly (B) and an increased proportion of fibrocyte precursor cells (define >8.4% as high) (C). **P* < 0.05, ***P* < 0.01.

**Supplementary Figure 2.** No significant changes in hemoglobin (A), platelet count (B), or spleen weight (C) were observed in *CHI3L1^‒/‒^* mice after Rom administration.
